# Red Tide Algal Toxin-Induced Intestinal Injury in Marine Fish: Mechanisms, Gut Microecology, and Aquaculture Health Risk Assessment

**DOI:** 10.3390/vetsci13101012

**Published:** 2026-09-25

**Authors:** Lingxiao Hang, Haihua Xu, Yanzhe Ding, Peipei Chen, Xingang Song, Dongsheng Shi, Zhongwei Chen

**Affiliations:** Nantong Marine Center, Ministry of Natural Resources, Nantong 226000, China; hlx20241208@163.com (L.H.); xuhh@ecs.mnr.com (H.X.); dingyanzhent@sina.com (Y.D.); peipychan@163.com (P.C.); songxingang19730116@hotmail.com (X.S.)

**Keywords:** red tide algal toxins, marine fish, intestinal injury, gut microecology, aquaculture health, risk assessment

## Abstract

Harmful algal blooms (HABs) can cause rapid, extensive mortality in exposed marine fish, often before fish-level diagnostic sampling can guide an emergency response. This review compares the toxin-specific evidence for intestinal injury caused by paralytic shellfish toxins, okadaic acid and Dinophysistoxins, brevetoxins, and fish-killing blooms, while distinguishing direct evidence in marine fish from evidence in other models. Intestinal barrier disruption, inflammation, and microbiome changes are valuable for explaining sublethal exposure, survivor recovery, and post-event attribution. They are not proposed as operational triggers during an acute fish kill. Practical management instead depends chiefly on site-specific environmental surveillance before exposure, predefined emergency actions when a bloom approaches, and longer-term reduction in avoidable nutrient and waste inputs where these contribute to local bloom risk. A four-stage framework links environmental hazard, fish exposure, biological effects, and production or food-safety outcomes with quantitative indicators and explicit uncertainty at each transition. This revised scope places mechanistic intestinal toxicology within the ecological time scale and operational limits of marine aquaculture.

## Simple Summary

Harmful algal blooms can cause widespread deaths of farmed marine fish so quickly that collecting and testing fish cannot guide an emergency response. This review examines how different algal toxins may damage the intestine, alter its protective barrier, and disturb the microorganisms that live there. Evidence differs among toxins and fish species, and results from laboratory cells or other animals cannot always be applied to marine fish. Understanding intestinal injury is most useful for studying repeated or less severe exposure, the recovery of surviving fish, and the causes of damage after a bloom. Practical protection depends on monitoring the environment before exposure, preparing feasible farm response plans, and reducing avoidable nutrient and organic waste inputs where these contribute to bloom risk. The review connects these management priorities with research on intestinal health and identifies the measurements and local validation needed before experimental findings can support decisions in aquaculture.

## 1. Introduction

Harmful algal blooms (HABs), commonly referred to as red tides, represent a significant and escalating environmental challenge in marine ecosystems worldwide. These phenomena are characterized by the rapid proliferation of certain microalgae species, often dinoflagellates and cyanobacteria, which produce potent biotoxins capable of causing extensive harm to aquatic organisms, human health, and economic activities such as fisheries and aquaculture. The frequency, intensity, and geographic distribution of HABs have increased markedly in recent decades, a trend attributed to a combination of anthropogenic nutrient enrichment, climate change, and other environmental stressors [1]. Coastal regions, estuaries, and aquaculture sites are particularly vulnerable to these blooms, which can lead to mass mortalities of marine fauna, including fish, shellfish, and other invertebrates, thereby disrupting ecosystem structure and function [2].

HAB-associated compounds such as okadaic acid (OA), domoic acid (DA), saxitoxins (STXs), microcystins (MCs), and prymnesins differ in source, route of exposure, and primary action. For orally acquired toxins, the gastrointestinal tract is a potential site of uptake and local injury; gill contact and other routes can be more important during an acute fish-killing event [3,4]. Marine-fish intestinal evidence remains less extensive than evidence for systemic toxicity or for effects in other experimental models. The fish gut also hosts microbial communities involved in nutrition and immune function, making it relevant to sublethal and post-event studies [5].

The strongest directly relevant gut microbiota evidence in this review comes from OA exposure in marine medaka, where changes in intestinal community composition and inflammatory readouts were measured [5]. A separate study exposed zebrafish larvae to ruptured *Karenia mikimotoi* cells and found intestinal barrier and infection-related effects; this freshwater model is useful for comparison but does not reproduce an acute marine-farm event [6]. Other experimental systems, including sea urchins exposed to OA, can suggest mechanisms but should not be treated as direct evidence of marine-fish intestinal injury [7]. The direction of any microbiota–injury relationship remains to be tested.

HABs affect aquaculture through several distinct mechanisms, including toxin exposure, gill damage, oxygen depletion, and other bloom-associated stressors; some fish-killing events progress too rapidly for intestinal sampling to change the outcome [8,9]. Nutrient enrichment can favor some blooms, but marine HAB causation varies by species and location [10]. Reducing controllable nutrient and organic-waste discharge is therefore a long-term, site-specific preventive measure, not a guaranteed way to stop any particular bloom. Intestinal pathology remains relevant for sublethal or recurrent exposures, survivors, and post-event diagnosis, whereas pre-event monitoring and contingency planning are the realistic basis for operational decisions.

Field events illustrate why ecological timing must be part of a fish-health review. In southern Chile, farm records linked harmful algal cell abundance to abnormal salmon behavior, while the 2016 *Pseudochattonella* event caused very large losses and prominent gill injury; mitigation effectiveness varied with the bloom and site [11,12]. A British Columbia analysis also identified HABs and low oxygen among major reported causes of farmed salmon mortality events [13]. Nutrient effects are equally context-dependent: a Chesapeake Bay time-series study of *Karlodinium veneficum* implicated both nutrient inputs and particulate prey, supporting source-specific investigation rather than a universal nutrient threshold [14].

Omics methods can characterize responses to HAB-related compounds, but the organ and host model must be specified. Transcriptomic studies of marine medaka exposed to OA provide fish-specific developmental and molecular data [15], while multi-omics studies of microcystin-LR in Nile tilapia examine the liver rather than marine-fish intestine [16]. Environmental molecular and chemical analyses can characterize bloom species and their products, as illustrated by work on *Prymnesium parvum* [17]. These methods are valuable for mechanism discovery and retrospective investigation, but they do not establish a rapid intestinal diagnostic test for farm use.

Environmental surveillance can provide useful lead time when a locally validated warning system exists. Algal identification, cell abundance, toxin analysis, dissolved oxygen, temperature, and current or wind forecasts are more actionable before exposure than intestinal tissue assays [18,19,20,21]. Once rapid mortality begins, the priority is to follow the farm and authority response plan, document environmental conditions, and protect unaffected stock where feasible; tissue collection is principally for subsequent investigation. Mechanistic studies can later help interpret sublethal injury and refine species-specific thresholds, but dose–response data and validated field cutoffs remain limited. Bloom dynamics also depend on local hydrography and multiple nutrient sources, so management should be based on site-specific ecological evidence rather than a universal intestinal biomarker threshold.

This review therefore separates three uses of the evidence: pre-event environmental prevention and warning, time-critical response to an emerging bloom, and post-event or sublethal assessment of fish injury. We synthesize intestinal mechanisms and microbiome findings by toxin class, evaluate the strength of marine-fish evidence, and propose a conditional framework for research and retrospective risk analysis. The framework is not intended to imply that fish can be sampled and a mechanistic risk score calculated quickly enough to prevent mortality during a severe HAB.

## 2. Types, Sources of Red Tide Algal Toxins, and Exposure Pathways in Marine Fish

### 2.1. Major Types of Algal Toxins: Chemical Structures and Toxicological Characteristics

Paralytic shellfish toxins (PSTs), including saxitoxin (STX), are produced by dinoflagellates such as *Alexandrium* spp. Their established primary action is blockade of voltage-gated sodium channels [22]. PST extracts from *Alexandrium* caused changes in glutathione metabolism, transporter activity, and lysosomal membrane stability in isolated rainbow-trout intestinal tissue and cells; this is experimental intestinal evidence, although rainbow trout is not a marine fish [23]. Gonyautoxins also induced cytotoxicity and apoptosis in human Caco-2 intestinal cells [24]. These findings support investigation of intestinal effects but do not show that gut injury is the dominant cause of mortality in marine-fish blooms.

Okadaic acid (OA) and dinophysistoxins (DTXs), produced by dinoflagellates such as *Dinophysis* and *Prorocentrum*, inhibit protein phosphatases PP1 and PP2A. Altered phosphorylation can disrupt epithelial structure and barrier function. Fish-focused evidence has been reviewed [3], while direct tight-junction experiments cited here used human intestinal cells [25]. OA-associated microbiota changes have been observed in marine medaka [5]. The evidence therefore supports an intestinal research focus for OA/DTXs, but mechanisms demonstrated in human cells should not be presented as established in every marine fish species.

Brevetoxins, produced by *Karenia brevis*, are lipophilic polyether compounds that promote activation of voltage-gated sodium channels. Fish exposure and accumulation have been documented, including toxin in viscera and muscle after experimental exposure [26]. Primary neurotoxic and waterborne effects may dominate an acute event. Direct evidence for a distinct brevetoxin-induced intestinal mechanism in marine fish is much thinner than the evidence for OA-associated gut responses; tissue accumulation alone does not establish intestinal barrier injury.

The toxin classes should not be pooled into a single intestinal mechanism. PSTs block voltage-gated sodium channels and are primarily associated with acute neurotoxicity; direct evidence for a specific marine-fish intestinal pathway is limited. OA and DTXs inhibit PP1/PP2A, making epithelial phosphorylation, barrier permeability, and gut microbiota changes the best-supported intestinal mechanisms among the groups reviewed [3,5]. Brevetoxins keep sodium channels active, and systemic or gill-mediated injury may dominate before gut effects can be evaluated. Fish-killing *Karenia* blooms can also involve mechanisms that are not attributable to one purified toxin. Microcystin-LR and cylindrospermopsin are included only as mechanistic comparators, largely from freshwater or non-fish models, and cannot be treated as equivalent evidence for marine red tide fish kills. Table 1 contrasts targets, exposure routes, observed gut endpoints, and the limits of each evidence base.

Additional studies clarify the boundaries of toxin comparison. *Karenia selliformis* toxicity has been examined in marine medaka, but whole-alga exposure does not identify one intestinal toxin mechanism [27]. OA effects on anchoveta larvae demonstrate a marine-fish developmental and sensory endpoint, whereas OA/DTX effects in *Artemia* are invertebrate evidence and should be kept separate from fish gut outcomes [28,29].

### 2.2. Main Exposure Pathways of Marine Fish to Algal Toxins

Exposure route depends on the toxin and bloom. Fish can ingest toxic cells or contaminated prey, and experimental intestinal uptake of PSTs has been demonstrated in rainbow-trout preparations [23]. Brevetoxin accumulation in fish after feeding or waterborne exposure has also been reported [26]. These observations support oral exposure as one possible route; they do not justify assigning the same dominant route to every harmful species or farmed fish.

Water-column exposure can directly affect fish during a bloom, including contact with the gills and other external surfaces. Pathology of fish exposed to a *Karenia mikimotoi* bloom illustrates the importance of whole-bloom injury [8]. Dissolved algal toxins have been measured in coastal waters [30], but their presence alone does not establish gill absorption, biliary excretion, and enterohepatic recirculation in a given fish species. Those toxicokinetic steps require toxin-specific measurements.

Trophic transfer and tissue accumulation occur for some HAB toxins, but biomagnification should not be assumed across toxin classes. Experimental and field evidence shows that brevetoxins can accumulate in fish tissues [26], and seafood-vector risks vary by toxin and fish species [4]. For aquaculture, repeated exposure to bloom material or contaminated feed is a plausible sublethal scenario, although the frequency and intestinal dose must be documented at the site. This distinction matters because acute waterborne fish kills and prolonged dietary exposure require different assessment methods.

Measured exposure can persist outside a visibly dense bloom. A Scottish survey detected domoic acid and PST analogues in the viscera of multiple fish species, documenting trophic exposure without establishing intestinal disease in those fish [31]. The persistence of dissolved OA and DTX-1 in seawater varies with microorganisms and environmental conditions, which matters for sampling time and exposure reconstruction [32]. Regional surveys of marine toxin formation and distribution provide context for selecting local analytes rather than assuming a fixed universal toxin panel [33].

### 2.3. Metabolism and Distribution Characteristics of Algal Toxins in Marine Fish

Absorption, transformation, and tissue distribution differ among HAB toxins and fish species; a single metabolic pathway cannot be assigned to all classes. Experimental work demonstrates intestinal uptake and effects of PSTs in rainbow trout preparations [23], while brevetoxins have been measured in fish viscera and muscle after exposure [26]. OA transformation in the gut lumen has been studied in rats [34], but those findings require testing before application to marine fish.

Biliary elimination and microbial transformation may be relevant for particular compounds, but the cited evidence does not establish enterohepatic recycling as a general mechanism for HAB toxins in marine fish. Studies should measure parent toxins and metabolites in water, gut contents, bile, and tissues over time before proposing a fish-specific recycling pathway. Evidence from rat gut-lumen OA metabolism provides a hypothesis rather than direct proof in marine fish [34].

Domoic acid offers an example of why toxicokinetic claims must remain toxin-specific. In coho salmon, orally administered domoic acid entered plasma rapidly and was detected in several tissues, while renal and biliary clearance appeared important; these findings do not establish the same clearance pathways for OA, PSTs, or brevetoxins [35]. Fish-consumption studies of microcystins address a different, mainly freshwater, food-safety context and should be treated as a comparator rather than evidence of marine-fish red tide risk [36].

Species and life stage can alter the response to a harmful alga. For example, early-life exposure to *Alexandrium tamarense* has been examined in *Larimichthys crocea* [37]. Such studies support species-specific assessment, but the available references do not substantiate a general claim that flatfish clear STX more slowly than perciform fish. Comparative toxicokinetic and intestinal studies are still needed.

In summary, fish exposure and tissue distribution are toxin- and species-dependent. Evidence for intestinal uptake should be separated from evidence for systemic accumulation, and results from mammals or freshwater fish should be labeled as comparative. Table 1 summarizes the primary targets, observed intestinal endpoints, models, and evidence limits for the toxin groups discussed.

**Table 1:** Comparative characteristics of representative HAB toxins and their relevance to intestinal injury in fish.

Toxin Class/Representative Toxin	Principal HAB Producers	Primary Molecular Target/Action	Dominant Exposure Route	Intestinal or Gut-Related Effects Emphasized in the Review	Representative Evidence Model	Interpretive Value/Evidence Boundary	References
Paralytic shellfish toxins (PSTs): STX, GTXs	*Alexandrium* spp.	Blockade of voltage-gated Na+ channels; disturbance of ion homeostasis; GTXs can additionally induce oxidative stress and apoptosis in intestinal epithelial cells.	Ingestion of toxic cells/contaminated prey; dissolved-toxin exposure; trophic transfer.	Experimental PST extracts altered epithelial glutathione metabolism, ABCC transport and lysosomal stability in rainbow trout; GTX-associated cytotoxicity was reported in human cells.	Isolated rainbow-trout middle intestine and enterocytes; human Caco-2 cells; early-life *Larimichthys crocea* exposed to *Alexandrium*.	Direct intestinal evidence includes a freshwater fish preparation; marine-fish intestine-specific mechanisms remain incompletely characterized.	[22,23,24,37]
Diarrhetic shellfish toxins (DSTs): OA, DTXs	*Dinophysis* spp.; *Prorocentrum* spp.	Inhibition of PP1/PP2A, protein hyperphosphorylation, cytoskeletal disruption and tight-junction destabilization.	Oral uptake is dominant; lipophilicity favors tissue retention and repeated intestinal exposure.	Increased epithelial permeability; disruption of claudins, occludin and ZO proteins; cytotoxicity, inflammation and microbiota dysbiosis.	Marine medaka *Oryzias melastigma*; *Caco-2* cells; fish-focused literature synthesis.	Best-developed evidence base in the review for linking a marine phycotoxin to gut barrier and microbiota changes.	[3,5,25,38]
Brevetoxins (PbTxs)	*Karenia brevis*	Persistent activation of voltage-gated Na+ channels, membrane depolarization and excitotoxicity.	Water-column exposure through gills plus ingestion of contaminated prey/particles.	Fish tissue accumulation is documented; a distinct marine-fish intestinal barrier mechanism is not established.	Fish and seafood-vector evidence summarized in marine-toxin reviews.	Compared with OA, the manuscript identifies a relative shortage of direct mechanistic studies focused specifically on marine-fish intestine.	[4,26]
*Ichthyotoxic Karenia* bloom products/*K. mikimotoi*-associated toxicity	*Karenia mikimotoi*	Multifactorial ichthyotoxicity associated with bloom exposure rather than a single universally established toxin mechanism.	Direct water exposure and ingestion during blooms.	Epithelial injury and barrier disruption; increased susceptibility to *Vibrio* infection has been reported in fish models.	Wild/cultured fish pathology; zebrafish-larva intestinal-barrier/infection model.	Highly relevant to real bloom-associated fish injury, but toxin-specific causality can be difficult to separate from whole-bloom effects.	[6,8]
Microcystins: MC-LR (HAB comparator)	*Microcystis* spp.	PP1/PP2A inhibition, oxidative stress, inflammatory signaling and gut-liver-axis disruption.	Primarily oral exposure to toxin-containing cyanobacterial material.	Microbiota dysbiosis, altered intestinal metabolism, increased LPS/inflammation and downstream hepatic immunotoxicity.	*Danio rerio*; *Cyprinus carpio*; mouse mechanistic models.	Not a classical marine red tide toxin, but provides strong comparative mechanistic evidence for microbiota-mediated intestinal and systemic injury.	[39,40,41]
Cylindrospermopsin (HAB comparator)	*Cylindrospermopsis/Raphidiopsis* spp.	Protein-synthesis inhibition and oxidative/cellular stress with secondary inflammatory effects.	Oral exposure to contaminated water or food.	Gut dysbiosis, increased Claudin-2-associated leakiness and gut-derived hepatic inflammation/fibrotic signaling.	Subchronic oral mouse model.	Useful for the gut-liver-axis concept, but evidence is not marine-fish-specific and should be interpreted as mechanistic support.	[42,43]

Abbreviations: STX, saxitoxin; GTXs, gonyautoxins; OA, okadaic acid; DTXs, dinophysistoxins; PP1/PP2A, protein phosphatases 1/2A; ZO, zonula occludens; LPS, lipopolysaccharide. MC-LR and cylindrospermopsin are retained as HAB mechanistic comparators because they are used extensively in later sections of the manuscript.

## 3. Damage of Red Tide Algal Toxins to Intestinal Tissue Structure and Barrier Function in Marine Fish

### 3.1. Morphological Characteristics of Intestinal Histopathological Damage

Intestinal histopathology can document epithelial injury after exposure, but lesion patterns and timing should be interpreted by toxin class and fish species. OA exposure in marine medaka has been associated with intestinal histological changes and inflammation, while a fish-focused review summarizes the broader but still limited OA/DTX evidence [3,5]. Necrosis and epithelial sloughing have also been observed in fish affected by a *Karenia mikimotoi* bloom, although attribution to a single toxin or to an intestine-first mechanism is uncertain [8]. Villus shortening, goblet-cell changes, congestion, and inflammatory infiltration are descriptive endpoints where measured, not HAB-specific diagnoses. Chronic fibrosis or impaired growth requires longitudinal evidence and should not be inferred from acute lesions. During rapid mass mortality, histology is primarily a post-event investigative tool.

### 3.2. Changes in Intestinal Mucosal Barrier Permeability and Regulatory Mechanisms

Tight-junction disruption is a plausible mechanism of increased intestinal permeability, particularly for OA and DTXs because phosphatase inhibition changes epithelial phosphorylation and cytoskeletal regulation. OA effects on tight-junction proteins have been demonstrated in human intestinal cell models [25], while marine-medaka evidence documents intestinal inflammation and microbiota changes without establishing that exact junction sequence [5]. Expression of claudins, occludin, and ZO proteins, together with functional permeability assays, can test the pathway in controlled fish studies. The same signaling sequence should not be assumed for PSTs or brevetoxins, whose primary sodium-channel actions differ. Cell-culture findings do not establish a field-ready diagnostic threshold for marine fish.

### 3.3. Molecular Mechanisms of Intestinal Epithelial Cell Injury

Oxidative stress, apoptosis, inflammatory signaling, and altered epithelial repair are candidate downstream processes in toxin-exposed intestinal models. For gonyautoxins, oxidative stress and apoptosis have been reported in human Caco-2 cells [24]; these data do not prove the same response in living marine fish. OA-associated barrier effects and microbiome changes have a stronger fish-specific basis, but proposed ER stress, NLRP3 activation, autophagy defects, and stem-cell suppression require direct validation for each toxin, dose, and species. Figure 1 therefore separates established primary targets from proposed downstream intestinal pathways. Acute fish-killing effects may arise from gill or systemic injury before such pathways can be measured.

Evidence from fish-killing algae also points to competing non-intestinal mechanisms. A comparative gill-cell assay found species-dependent effects of harmful algae, including roles for cell lysis and oxidative products [44]. Acute *Karenia mikimotoi* exposure produced oxidative stress and DNA damage in red sea bream gills, and an experimental modified-clay study in marine medaka linked intact-cell density to gill injury and rapid death [45,46]. OA plus microplastic exposure has been studied in intestinal cell and fermentation systems, but those co-exposure findings are not direct evidence of field injury in marine fish [47].

## 4. Role and Mechanisms of Gut Microecology in Algal Toxin-Induced Intestinal Injury

### 4.1. Effects of Algal Toxins on Gut Microbiota Composition and Diversity

The best directly relevant marine-fish evidence in this review concerns OA-associated changes in intestinal microbiota of marine medaka [5]. Microbiome shifts are also reported in fish and other taxa exposed to different HAB-related compounds, but responses cannot be generalized across PSTs, brevetoxins, OA/DTXs, and fish-killing blooms. Alpha diversity and the abundance of opportunistic taxa are descriptive endpoints rather than toxin-specific signatures. Diet, age, salinity, exposure duration, and baseline microbiota can alter the measured community. Reports from cyanobacterial blooms and non-marine systems provide comparative hypotheses, not proof that the same taxa or pathways occur during a marine HAB fish kill [39,48].

### 4.2. Causal Relationship Between Gut Microbiota Dysbiosis and Intestinal Injury

A causal microbiota contribution to intestinal injury remains a hypothesis for most marine-fish HAB toxin exposures. OA-associated microbial changes and barrier effects have been reported in marine medaka [5], but a simultaneous change does not establish whether dysbiosis drives injury, follows epithelial damage, or reflects both. Microbial metabolites and inflammatory pathways such as LPS-related signaling are mechanistically plausible and can be tested with controlled perturbation and rescue experiments. Evidence from probiotic supplementation or microbiota transfer in other hosts should not be presented as a demonstrated farm-level treatment for acutely exposed marine fish. Figure 2 depicts candidate interactions and their evidence boundary.

### 4.3. Role of Microbiota–Host Co-Metabolism in Modulating Algal Toxin Toxicity

Microbial transformation could alter toxin bioavailability, but specific reactions and their net effects in the marine-fish gut need direct measurement. Reports of toxin binding or removal by bacteria, including work in non-fish systems, identify candidate mechanisms rather than established protection during a HAB [49]. Host–microbiome metabolite signaling may modify inflammation after sublethal exposure, yet these pathways are not interchangeable among toxin classes. Paired toxin-metabolite measurements and experimentally manipulated microbiota would be needed to distinguish detoxification from simple adsorption or correlated community change.

Comparative interpretation: the marine-medaka OA literature offers direct evidence of toxin-associated microbiota change, although causality and direction of effect require further testing [5]. Equivalent toxin-specific gut microbiome evidence in marine fish is much thinner for PSTs and brevetoxins. Microcystin and cylindrospermopsin studies illustrate possible host microbiome pathways but cannot establish that the same taxa, metabolites, or responses occur during a marine red tide event.

The comparator literature also varies in outcome and host. Acute cyanotoxin poisoning in mice produced relatively modest microbiome composition changes but measurable metabolic shifts, whereas longer microcystin exposure in frogs altered the gut community [50,51]. A tadpole intervention study explored microbiota-linked protection from MC-LR, and OA/DTX experiments in *Artemia* examined a separate invertebrate toxicity system [29,52]. None of these models validates a probiotic rescue strategy for marine fish during a rapid HAB.

## 5. Systemic Pathological Effects and Immune Dysregulation Induced by Algal Toxins via Intestinal Injury

### 5.1. Spread of Gut-Derived Inflammation to Systemic Circulation

Intestinal barrier failure may contribute to systemic inflammation after sustained exposure, but the evidence varies substantially among toxins and hosts. MC-LR effects on the gut-liver axis have been studied in common carp, and cylindrospermopsin-related changes have been reported in mice [40,42]. These studies are mechanistic comparators rather than direct evidence that gut-derived inflammation drives acute marine-fish deaths during a red tide. For OA/DTX exposure, a gut-mediated contribution is more biologically aligned with the known epithelial effects; for PSTs and brevetoxins, primary neurotoxic or gill-associated processes may dominate. Claims of systemic cytokine spread, hepatic detoxification failure, and multi-organ injury require toxin- and species-specific confirmation.

### 5.2. Dysregulation of the Intestinal Immune System

Mucosal immune responses may change after intestinal injury, but evidence for a common pattern of IgM-, IgT-, T-cell-, and antimicrobial-peptide changes across marine HAB toxin classes is insufficient. Findings from microcystin, amphibian, mammalian, and cell studies can guide experiments but cannot be transferred directly to marine fish. Future fish studies should pair immune phenotyping with measured toxin dose, intestinal pathology, and infection status to determine whether immune changes are caused by the toxin or by secondary stress. Such measurements are research endpoints for sublethal exposure and survivors rather than practical markers during a rapid fish-killing event.

### 5.3. Dysregulation of the Neuro–Immune–Gut Axis

PSTs and brevetoxins differ fundamentally in sodium-channel action: PSTs block channel conduction, whereas brevetoxins promote persistent activation. Both can cause acute neural or systemic dysfunction, but direct evidence that a neuro-immune-gut axis mediates their intestinal effects in marine fish remains limited. Enteric neurotransmitter and immune signaling pathways offer hypotheses for controlled studies, not a proven explanation of field mortality. Any putative gut-axis effect should be evaluated against the competing time course of gill injury, hypoxia, and primary neurotoxicity in each bloom.

Enteric glial cells may participate in phycotoxin responses in an intestinal tri-culture model, which provides a mechanistic hypothesis rather than an observed neuro-immune pathway in marine fish [53]. Repeated MC-LR exposure has also been linked to gut microbiota and systemic inflammation in a mammalian study; again, the host, toxin, and exposure window differ from an acute marine bloom [54].

Comparative interpretation: an intestinal contribution to systemic inflammation is plausible after sustained barrier injury, especially in OA/DTX exposure models, but acute mortality from sodium-channel toxins or fish-killing bloom contact can precede this sequence. The MC-LR and cylindrospermopsin studies below are comparative evidence from different taxa and environments. They should not be used to infer a universal gut-mediated pathway for all marine HAB classes.

## 6. Detection Techniques, Biomarkers, and Risk Assessment Models for Intestinal Injury in Seawater Fish

### 6.1. Precise Diagnosis of Intestinal Injury Based on Multi-Omics Technologies

Multi-omics methods are most useful for mechanism discovery and retrospective analysis rather than immediate diagnosis during a fast-moving fish kill. OA-exposed marine medaka provide fish-specific transcriptomic evidence, whereas microcystin-LR studies in zebrafish and OA studies in human liver cells provide comparative, not marine-fish intestinal, omics evidence [15,39,55]. These readouts are often nonspecific and require species-, toxin-, dose-, tissue-, and time-matched controls. Histopathology provides interpretable confirmation of established intestinal injury but requires destructive sampling. No intestinal omics signature cited here has been prospectively validated to trigger a time-critical farm response. In summary, locally validated environmental monitoring offers the most promising early warning before exposure, whereas histopathology is best suited to confirming injury in survivors and multi-omics remains a research tool for mechanism discovery.

### 6.2. Molecular Biomarkers Suitable for On-Site Screening

For the earliest actionable warning at a marine farm, locally calibrated environmental surveillance is currently more promising than a fish intestinal biomarker: harmful-taxon identification, toxin testing where appropriate, oxygen measurement, and hydrodynamic forecasts can identify exposure before severe injury [19,20,21]. Existing on-farm molecular workflows include systems developed for other aquaculture sectors and require validation for finfish sites. Rapid field tests must be evaluated against the local harmful species and should be interpreted with confirmatory laboratory methods when decisions allow time. Targeted fish assays, such as barrier-gene expression or histology, may help assess surviving stock after sublethal exposure, but they are invasive or too slow for most acute events. Circulating miR-122 is primarily a liver-injury marker in the cited fish study and should not be presented as an intestine-specific field test [56]. Microbiome profiling is sensitive to diet, age, salinity, and husbandry and presently belongs in research or post-event investigation. Table 2 distinguishes exposure warning from confirmation of intestinal injury. Surveillance methods address complementary questions. Satellite imagery has been used to map dinoflagellate blooms near aquaculture production areas, passive toxin-adsorption devices can track lipophilic toxins, and ensemble forecasting can improve near-term bloom risk estimates [57,58,59]. None directly diagnoses intestinal injury. Local verification of harmful species, depth distribution, toxin profile, and warning lead time remains necessary before these measurements can trigger a farm action.

### 6.3. Four-Stage Framework for Prevention, Response, and Post-Event Assessment

A four-stage causal framework is proposed: (1) environmental hazard, (2) fish exposure, (3) biological effect, and (4) production and food-safety consequence (Figure 3). The first stage records harmful species, cell abundance, measured toxin where applicable, dissolved oxygen, nutrient conditions, and forecast transport. The second estimates exposure at a specific farm from concentration, duration, water exchange, fish location, and feeding or gill-contact routes. The third records acute signs and mortality; intestinal histology, permeability, or microbiome measures are optional research or survivor endpoints, not prerequisites for emergency action. The fourth records fish losses, growth or recovery of survivors, and edible-tissue testing under applicable food-safety rules. At each transition, the quantitative output of the preceding stage must be linked to the next by a locally estimated relationship: for example, a time series of cell or toxin concentration and current direction informs predicted farm exposure; an exposure–response curve informs the probability of mortality or sublethal injury; and observed mortality and tissue contamination inform economic and food-safety consequences. Such relationships cannot be assumed transferable across species, sites, and toxin classes. If sufficient site-specific data exist, Monte Carlo simulation can repeatedly sample uncertain inputs such as concentration, duration, and response curve parameters to produce a distribution of predicted losses rather than a single value; this is a research and planning tool, not a real-time rescue calculation. Without validated dose–response data, report measured indicators and uncertainty instead of calculating a hazard quotient or margin of exposure from inappropriate endpoints. Operationally, the framework has three decision windows: before a bloom, reduce controllable nutrient and waste inputs and use environmental early warning; as a bloom approaches, implement a feasible, predefined farm response; after the event, investigate causation, assess survivors, and address product safety. A severe, rapidly lethal event may bypass the opportunity for fish-level sampling altogether.

## 7. Aquaculture Health Protection Strategies and Future Research Prospects

### 7.1. Prevention and Intervention Measures at the Aquaculture Management Level

The first management priority is prevention at the coastal system scale. Where local evidence links nutrient enrichment to harmful blooms, wastewater treatment, control of agricultural and urban runoff, feed efficiency, stocking practices, and management of farm effluent and organic waste can reduce avoidable inputs; their effect depends on species, hydrography, and other nutrient sources [10]. Farms should pair regional bloom surveillance with site-specific alert thresholds and written response plans before a bloom reaches the cages [9,21]. When warnings provide lead time, possible actions include pausing feeding, adjusting water intake in controllable systems, or moving stock only if this is technically and biologically feasible. These actions cannot be assumed effective after a severe bloom has reached open-water cages. During mass mortality, environmental measurements and coordinated emergency management take priority over intestinal biomarker sampling. Nutritional additives and probiotics remain experimental or supportive strategies for sublethal exposure and recovery; they cannot neutralize an acute fish-killing bloom, and evidence from non-marine or non-fish systems should not be presented as proof of farm-level protection.

An experimental modified-clay treatment reduced *Karenia mikimotoi* cell density and toxicity to marine medaka, but its efficacy and ecological effects require site-specific assessment before operational use [46]. The Chilean *Pseudochattonella* investigation found that some attempted farm mitigation measures were ineffective under that bloom’s conditions, underscoring the need to test contingency actions against the local species and water-column structure [12].

### 7.2. New Methods and Technologies in Algal Toxin Intestinal Toxicology Research

Fish-derived intestinal culture systems can help test barrier and cellular responses under controlled exposure conditions. Organotypic rainbow-trout intestinal platforms demonstrate that experimental design affects epithelial differentiation and may support future toxicology studies [62]. More complex co-culture and microfluidic systems are research prospects, but they require validation against intact-fish exposures before their results can be generalized to marine aquaculture. Mechanistic experiments should identify the toxin or bloom material, measure the delivered dose, and distinguish direct epithelial effects from responses to hypoxia, infection, and other bloom-related stressors. These approaches can strengthen causal interpretation; they are not established emergency tools for a rapidly developing fish kill.

### 7.3. Epidemiological and Ecological Risk Research Trends of Red Tide Algal Toxin Intestinal Toxicity

Future field research should test whether locally monitored harmful species, toxin concentrations, oxygen conditions, currents, and nutrient loading predict exposure early enough for farms to act [9,21]. This requires time-stamped event records that include alert lead time, actions taken, mortality, and environmental measurements. Nutrient-source apportionment and evaluation of effluent and wastewater remediation are important where eutrophication is implicated, while recognizing that bloom initiation and persistence also reflect physical conditions and species ecology. Intestinal pathology and microbiome sampling should be focused on sublethal exposures, survivors, and retrospective causal studies. Prospective validation is needed before any fish biomarker can be claimed as an early operational warning signal.

## 8. Conclusions

HABs can kill exposed marine fish rapidly through several ecological and physiological pathways, so a mechanistic assessment of intestinal injury cannot generally be completed in time to direct rescue during a severe event. The intestinal evidence reviewed here is most relevant to sublethal exposure, survivor health, and post-event attribution. OA and DTXs have the clearest reviewed link to intestinal barrier and microbiome changes, whereas evidence for other toxin classes is less direct or concerns different dominant mechanisms. These differences and evidence gaps should remain explicit in interpretation.

For aquaculture practice, the defensible sequence is to reduce avoidable nutrient and organic-waste inputs where they contribute to local bloom risk, monitor environmental hazards before exposure, prepare feasible farm-specific actions, and assess fish and seafood after an event. Quantitative risk models may assist planning when local exposure-response data exist; they should not be presented as an emergency decision tool without demonstrated lead time and validation. Future work should prioritize ecology-linked forecasting, field verification of intervention windows, and carefully controlled studies of intestinal effects in surviving marine fish.

## Figures and Tables

**Figure 1:**
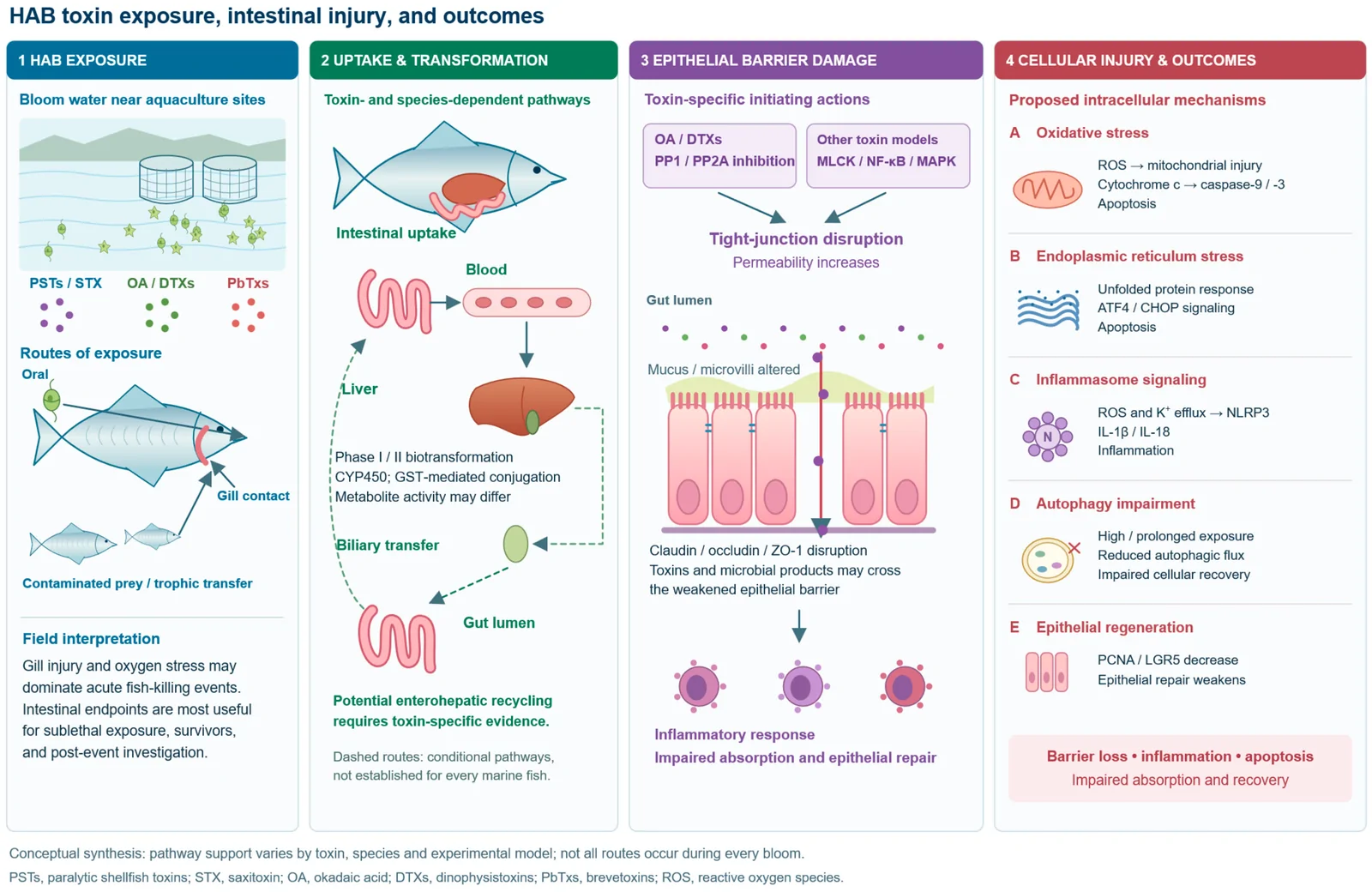
Conceptual pathways linking harmful algal bloom (HAB) exposure to intestinal and systemic injury in marine fish. The four numbered, color-coded sections represent exposure (blue), uptake and transformation (green), epithelial barrier damage (purple), and cellular injury and outcomes (red). Fish may encounter HAB cells and toxins through ingestion, gill contact, and trophic transfer. Solid arrows indicate the direction of the illustrated processes or proposed effects and do not independently establish causality. Dashed arrows indicate conditional pathways, including potential enterohepatic recycling, that require toxin- and species-specific evidence. In the exposure section, purple, green, and red dots represent PSTs/STX, OA/DTXs, and PbTxs, respectively; these colors identify toxin groups rather than toxicity levels. Intestinal uptake, hepatic biotransformation, and biliary transfer depend on the toxin and fish species. OA and DTXs inhibit PP1/PP2A, while studies of other toxins implicate MLCK, NF-κB, or MAPK signaling in epithelial barrier dysfunction. The illustrated barrier changes include altered mucus and microvilli, tight-junction disruption, and increased permeability. Within the rightmost section, labels A–E identify five proposed mechanisms: (A) oxidative stress; (B) endoplasmic reticulum stress; (C) inflammasome signaling; (D) autophagy impairment; and (E) reduced epithelial regeneration. The red × in D denotes impaired autophagic processing. Evidence varies among toxins, species, and experimental models; the diagram does not imply that all pathways occur during every bloom or that intestinal injury is the principal cause of acute fish mortality.

**Figure 2:**
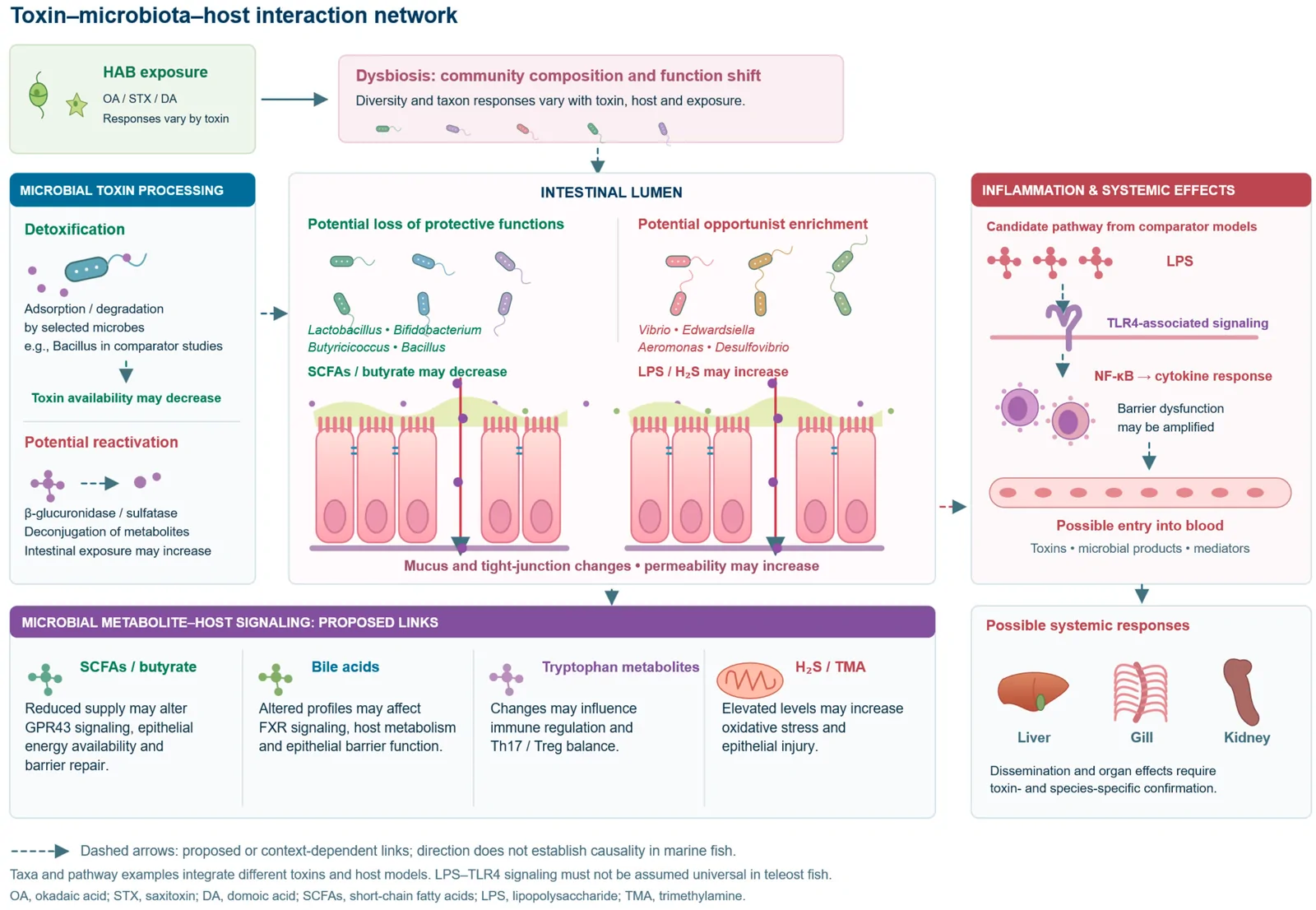
Conceptual toxin-microbiota-host interaction network. HAB exposure may alter gut microbial composition, microbial toxin transformation, and metabolite signaling. Representative taxa illustrate potential changes in protective functions and opportunist abundance; their responses depend on the toxin, host, and exposure conditions. The central epithelium depicts altered mucus, disrupted tight junctions, and possible passage of toxins and microbial products across a weakened barrier. Lower panels summarize proposed links involving short-chain fatty acids, bile acids, tryptophan metabolites, hydrogen sulfide, and trimethylamine. The right branch illustrates candidate inflammatory signaling and possible systemic effects. LPS–TLR4–NF-κB signaling is included as a mechanism from comparator models and should not be assumed universal in marine fish. Dashed arrows indicate proposed or context-dependent links. The network integrates findings from different experimental systems; causal relationships and their relevance to marine fish require toxin- and species-specific validation.

**Figure 3:**
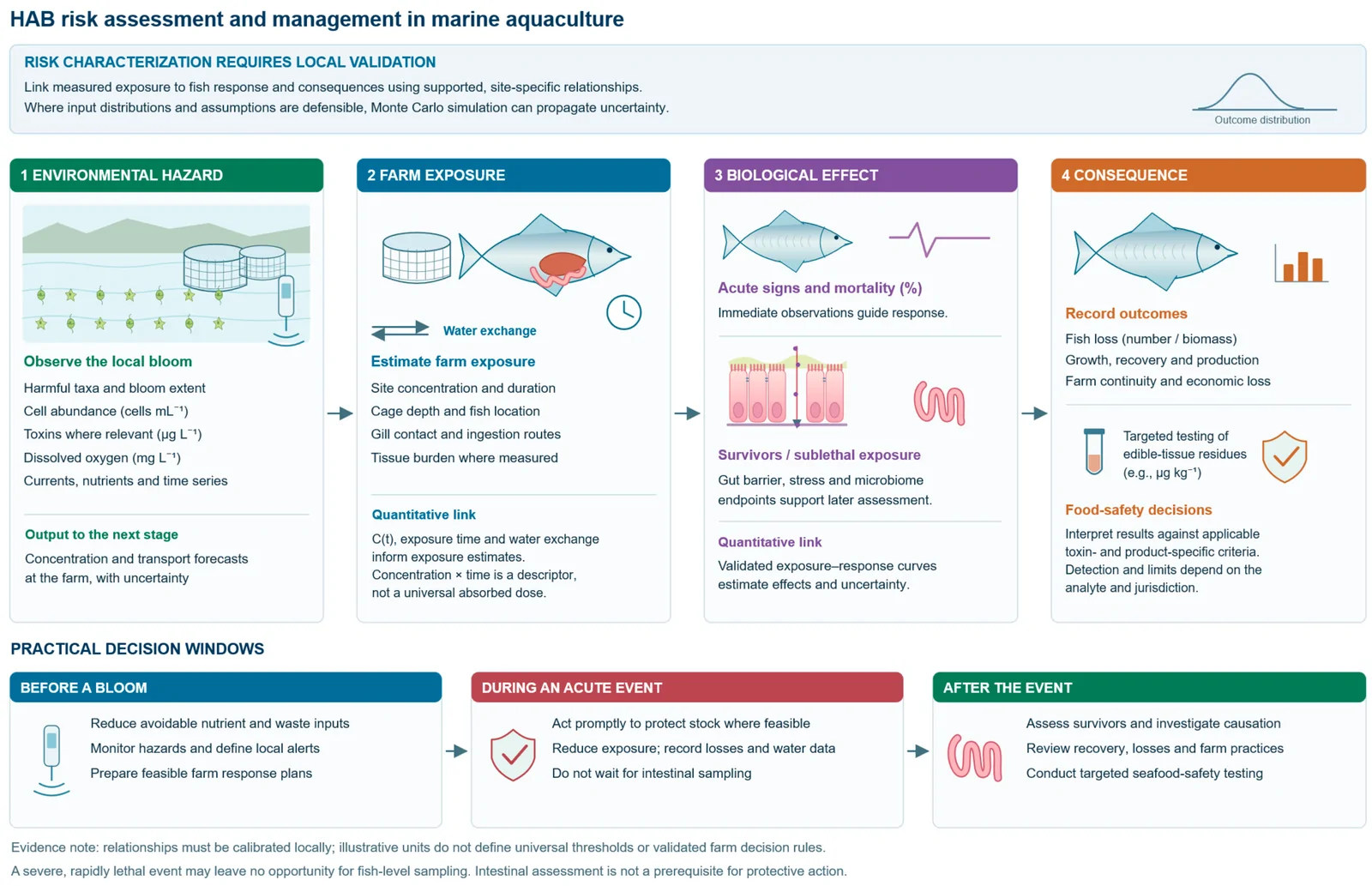
Four-stage harmful algal bloom (HAB) risk pathway and practical decision windows for marine aquaculture. The four numbered, color-coded stages represent environmental hazard (green), farm exposure (blue), biological effect (purple), and consequences (orange). Environmental hazard is described by harmful taxa, cell abundance, toxin concentration where relevant, dissolved oxygen, and transport. Farm exposure combines site concentration with duration, water exchange, and contact routes; the paired opposing arrows indicate water exchange. Biological effects include acute signs and mortality, with intestinal endpoints assessed mainly in survivors or after sublethal exposure. Consequences include fish loss, recovery, production, and targeted edible-tissue testing. Arrows connecting the four stages indicate the direction of the assessment pathway; quantitative relationships between stages require locally validated exposure and response data. The lower panels distinguish three management windows: before a bloom (blue), during an acute event (red), and after the event (green), with connecting arrows indicating their temporal sequence. Colors distinguish stages and management windows rather than quantitative risk levels. The schematic outcome distribution illustrates uncertainty in predicted consequences, not observed data; uncertainty analysis, including Monte Carlo simulation, is appropriate only when input distributions and assumptions are supported. Acute protective action does not depend on gut sampling.

**Table 2:** Comparative summary of diagnostic, biomarker, multi-omics, and surveillance approaches relevant to algal toxin-induced intestinal injury.

Assessment Category	Sample/Matrix	Representative Methods	Candidate Readouts	What the Readout Indicates	Main Advantage	Key Limitation for Field Translation	References
Histopathology and ultrastructure	Intestinal tissue	H&E staining; SEM/TEM; morphometry	Villus shortening/sloughing, epithelial necrosis, vacuolation, goblet-cell loss, microvillus disruption	Direct structural evidence of mucosal injury and loss of absorptive/barrier architecture.	High pathological interpretability and useful validation endpoint.	Destructive sampling; often detects established injury rather than the earliest molecular response.	[3,6,8]
Barrier-function molecular markers	Intestinal tissue or epithelial-cell models	qRT-PCR; Western blot; immunofluorescence; permeability/TEER assays	Claudins, occludin, ZO-1/ZO-2; permeability changes	Early disruption of epithelial tight-junction integrity and paracellular barrier function.	Sensitive and mechanistically linked to intestinal barrier failure.	Several marker relationships are supported by non-fish intestinal models; marine-fish validation remains necessary.	[25,38,60]
Oxidative-stress and apoptosis markers	Intestinal tissue; intestinal epithelial cells	ROS assays; caspase assays; biochemical analysis; targeted metabolite measurements	ROS, lipid-peroxidation products, caspase-3/9 activation, mitochondrial injury	Cellular stress and progression toward programmed epithelial-cell death.	Can detect toxic responses before gross pathology becomes severe.	Limited toxin specificity; responses overlap with hypoxia, infection, nutrition and other environmental stressors.	[24,39,61]
Gut-microbiota biomarkers	Intestinal content, mucosa or fecal material	16S rRNA sequencing; metagenomics; targeted qPCR	α-diversity, community structure, opportunistic taxa such as *Vibrio*, loss of beneficial/SCFA-associated taxa	Dysbiosis and altered microbial resilience associated with toxin exposure and intestinal inflammation.	High sensitivity to ecological disturbance and potential for repeated/non-lethal surveillance.	Strongly influenced by diet, age, salinity, temperature and husbandry; universal diagnostic cutoffs are not established.	[5,39,48]
Multi-omics pathway profiling	Intestinal tissue, gut lumen and related cell models	RNA-seq; metabolomics; proteomics; phosphoproteomics; integrative bioinformatics	DEGs, altered amino-acid/energy metabolism, phosphorylation networks, host-microbiome co-metabolic pathways	Mechanistic pathway discovery and identification of multi-marker signatures.	Broad molecular coverage and strong biomarker-discovery potential.	High cost and complex integration; some cited omics studies concern rat gut, zebrafish or human liver rather than marine-fish intestine.	[15,34,39,55]
Circulating miRNA/liquid-biopsy candidates	Plasma or serum	RT-qPCR/digital PCR	miR-122-5p and other exposure-responsive circulating miRNAs	Systemic tissue injury associated with algal-toxin exposure; potential minimally invasive early-warning signal.	Small sample volume, molecular stability and repeat-sampling potential.	Current fish evidence is stronger for systemic/liver injury than for intestine-specific diagnosis.	[56]
Environmental exposure surveillance	Seawater, algal cells and aquaculture-zone samples	SPE-HPLC-MS/MS; DNA biosensors; in situ monitoring; satellite/forecast models	Toxin concentration, toxin-producing algal abundance and bloom risk probability	Exposure likelihood before overt fish injury develops.	Supports proactive management, early warning and spatial risk mapping.	Measures exposure rather than intestinal injury; biological response biomarkers are still required for health diagnosis.	[18,19,20,21]

Abbreviations: H&E, hematoxylin and eosin; SEM/TEM, scanning/transmission electron microscopy; qRT-PCR, quantitative reverse-transcription PCR; TEER, transepithelial electrical resistance; DEGs, differentially expressed genes; SPE-HPLC-MS/MS, solid-phase extraction coupled with high-performance liquid chromatography-tandem mass spectrometry. The table distinguishes direct intestinal-damage readouts from exposure-surveillance tools to avoid treating environmental detection as a diagnosis of gut injury.

## Data Availability

No new data were created or analyzed in this study. Data sharing is not applicable to this article.
